# Genetic Variant in the CYP19A1 Gene Associated with Coronary Artery Disease

**DOI:** 10.1155/2015/820323

**Published:** 2015-03-16

**Authors:** Konstantina Bampali, Charalampos Grassos, Angeliki Mouzarou, Charalampos Liakos, Georgios Mertzanos, Klea Lamnissou, Dimitrios Babalis

**Affiliations:** ^1^Department of Genetics and Biotechnology, Faculty of Biology, University of Athens, 15784 Athens, Greece; ^2^Department of Cardiology, General Hospital “KAT”, 14561 Athens, Greece

## Abstract

The CYP19A1 gene encodes the enzyme aromatase, which is responsible for the biosynthesis of estrogens. The rs10046 polymorphism of CYP19A1 gene has been investigated in two studies on the occurrence of hypertension, but there are no studies on its correlation with coronary artery disease (CAD). We investigated 189 subjects who were hospitalized at “KAT” General Hospital of Athens and underwent coronary angiography. Of these, 123 were found with CAD with an average age of 60 years and constituted the patients group and 66 subjects with an average age of 58 years without damage in the coronary vessels and constituted the control group (healthy). The frequencies of genotypes CC, CT, and TT of rs10046 polymorphism are significantly different between the group of CAD patients and the control group (0.34, 0.48, and 0.18 versus 0.20, 0.48, and 0.32, resp., *P* = 0.034) as the frequency of C allele (0.58 versus 0.44, resp., OR = 1.771 and *P* = 0.010). We found similar results for men, but not for women (small sample). The results of this study show that the rs10046 (C/T) polymorphism of CYP19A1 gene exhibits correlation with CAD and that patients with C allele have an increased probability of manifesting the disease.

## 1. Introduction

It is obvious that geneticists would mainly focus on investigating the correlation of genes with ischemic cardiovascular disease since it is the first cause of morbidity and mortality worldwide [1, 2]. The CYP19A1 gene encodes the enzyme aromatase, which is responsible for the biosynthesis of estrogens. Mutations and other genetic changes in this gene can cause increased or decreased activity of aromatase [3, 4]. Genetic polymorphisms in CYP19A1 gene correlate with the activity of aromatase, sex hormone levels, and estrogen dependent conditions. However, it is unclear what molecular mechanisms are involved [5–9]. It has been demonstrated that both estrogens and aromatase are produced in vascular tissue, particularly in smooth muscle cells and endothelial cells [10, 11]. A recent study examined genetic changes of CYP19A1 gene to relate them to the differences according to gender in the outcome of cardiovascular disease. The outcome was more unfavourable for men compared with women [12].

The rs10046 polymorphism, a C/T single nucleotide polymorphism (SNP) located in the 3′ untranslated region (3′-UTR) of the CYP19A1 gene, has been related according to the number of T alleles either to trends of increased levels of circulating estrone and estradiol but no differences for androgens, either lower levels of several androgens but no differences for estrone or estradiol, or higher estrogen to androgen ratios [13]. However, the exact mechanism remains unclear. In cardiovascular system, the rs10046 polymorphism of CYP19A1 gene has been investigated in two studies on the occurrence of hypertension [14, 15]. However, there are no studies on its correlation with coronary artery disease (CAD). The purpose of this study is to investigate the possible association of rs10046 polymorphism of CYP19A1 gene with the early occurrence of CAD in Greek population.

## 2. Methods

We studied 189 subjects who were hospitalized for precordial pain investigation and underwent coronary angiography at the Cardiology Department of “KAT” General Hospital of Athens during 2012. All subjects were Greeks with no blood relationship. Of these, 123 were found with CAD with an average age of 60 years (81% men) and constituted the patients group. Coronary artery disease was defined as a luminal narrowing >70% of at least one vessel. The remaining 66 subjects with an average age of 58 years (78% men) exhibited no damage in the coronary vessels and constituted the control group (healthy). Subjects were defined hypertensive or diabetic if they were under treatment or their blood pressure was >140/90 mm Hg and fasting glucose >126 mg/dL, respectively. Smoking status was defined from current smokers. Ancient history of CAD was present in 19% of the patients' group and in 0% of the controls' group. Family history was considered positive for CAD patients if at least one first-degree relative was diagnosed with CAD by the age of 55 years. The data were obtained from medical files. All subjects provided written informed consent. The study was performed according to the Code of Ethics (Declaration of Helsinki).

**Figure 1:**
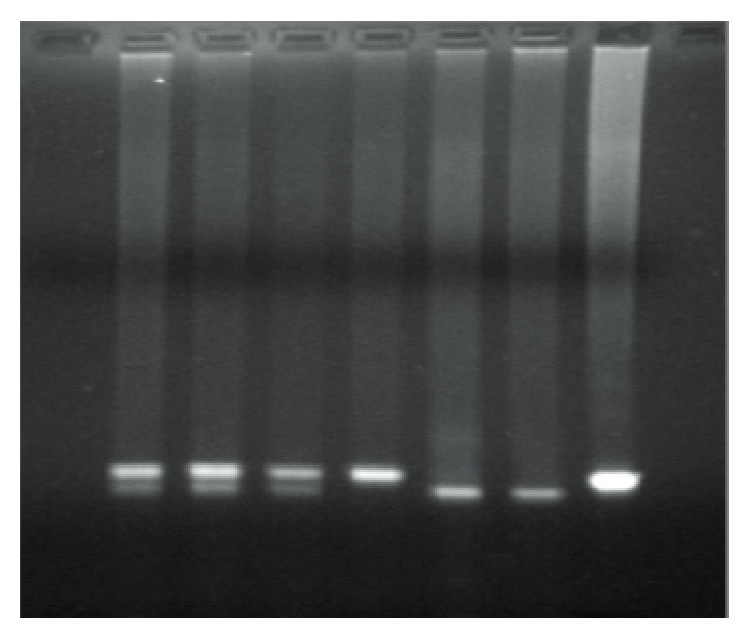
Electrophoresis of digestion product. Genotypes are visualized as follows: on lines 1, 2, and 3 CT, on line 4 TT, on lines 5, 6 CC, and on line 7 DNA sample control.

Blood was collected from all subjects after overnight fasting between 8 and 10 a.m. in supine position. Genomic DNA was extracted from leukocytes of peripheral blood samples by a standard salting out method [16]. The genotyping of the rs10046 polymorphism was determined by polymerase chain reaction-restriction fragment length polymorphism (PCR-RFLP) analysis. Restriction fragment length polymorphism or RFLP is a technique that exploits variations in homologous DNA sequences. In RFLP analysis, the DNA sample is broken into pieces (digested) by restriction enzymes and the resulting restriction fragments are separated according to their lengths by gel electrophoresis. The PCR amplification primers used were forward, 5′-CTG GAA CAC TAG AGA AGG CTG GTC AGT GC-3′, and reverse, 5′-GTT CTC TGG TGT GAA CAG GAG CAG ATC AC-3′. The PCR procedure was as follows: an initial denaturation step at 94°C for 2 min and then amplified for 35 cycles at 94°C for 30 sec, 57°C for 40 sec, and 72°C for 40 sec, followed by a final extension step at 72°C for 7 min. The PCR products were digested with the restriction enzyme Bsp1286 I (SduI), were separated by electrophoresis on 1,5% (w/v) agarose gel, and were visualized by ethidium bromide staining. The Bsp1286I restriction enzyme (New England, Biolabs, Hitchin, UK) recognizes GDGCH^∧^C sites (isoschizomers: SduI). The digestion of the PCR product by the restriction endonuclease Bsp1286 I (SduI) allowed discrimination between the alleles C and T of the CYP19A1 polymorphism. The 202 bp PCR product in the presence of C at 3′ UTR is cleaved into two fragments of 172 bp and 30 bp. The CC genotypes resulted in 172 bp and 30 bp, the CT genotypes in 202 bp, 172 bp, and 30 bp, and the TT genotypes in 202 bp digestion products (Figure 1). The subjects' genotypes were recorded as to the rs10046 polymorphism of the 3′ UTR region of the CYP19A1 gene and the frequencies of C and T alleles and CC, CT, and TT genotypes were specified in the two groups under study.

The genotyping of the rs10046 polymorphism was determined by polymerase chain reaction-restriction fragment length polymorphism (PCR-RFLP) analysis. The PCR amplification primers used were forward, 5′-CTG GAA CAC TAG AGA AGG CTG GTC AGT GC-3′, and reverse, 5′-GTT CTC TGG TGT GAA CAG GAG CAG ATC AC-3′. The PCR procedure was as follows: an initial denaturation step at 94°C for 2 min and then amplified for 35 cycles at 94°C for 30 sec, 57°C for 40 sec, and 72°C for 40 sec, followed by a final extension step at 72°C for 7 min. The PCR products were digested with the restriction enzyme Bsp1286 I (SduI), were separated by electrophoresis on 1,5% (w/v) agarose gel, and were visualized by ethidium bromide staining. The digestion of the PCR product by the restriction endonuclease Bsp1286 I (SduI) allowed discrimination between the alleles C and T of the CYP19A1 polymorphism. The 202 bp PCR product in the presence of C at 3′ UTR is cleaved into two fragments of 172 bp and 30 bp. The CC genotypes resulted in 172 bp and 30 bp, the CT genotypes in 202 bp, 172 bp, and 30 bp, and the TT genotypes in 202 bp digestion products (Figure 1). The subjects' genotypes were recorded as to the rs10046 polymorphism of the 3′ UTR region of the CYP19A1 gene and the frequencies of C and T alleles and CC, CT, and TT genotypes were specified in the two groups under study.

### 2.1. Statistical Analysis

Continuous variables are presented as mean ± SD and categorical variables as observed number (*n*, %). Unpaired Student's *t*-test was used to evaluate differences in continuous variables once normality was demonstrated (Kolmogorov-Smirnov or Shapiro-Wilk test); otherwise, a nonparametric test (Mann-Whitney) was used. Differences for categorical variables were analyzed with chi-square test (or Fisher's exact test, if applicable). Differences in genotype frequencies between CAD patients and controls were evaluated using the chi-square analysis for homogeneity. Differences in allele frequencies were tested for statistical significance at the 95% confidence interval using Fisher's exact test. A *P* value less than 0.05 was considered statistically significant. The odds ratio (OR) was used as a measure of the strength of the association between allele frequencies and CAD. Data analysis was performed with IBM SPSS 19 statistical software (2010, IBM Corporation, Route 100 Somers, NY 10589, USA).

## 3. Results

The clinical characteristics of the group of CAD patients and the control group are shown in Table 1. These are two similar groups, not statistically different from each other in terms of age, gender, hypertension, smoking, family history of CAD, and lipid levels. A percentage of 19% of the group of CAD patients reported ancient history of CAD, while control subjects did 0%.

The complete experimental results for the group of CAD patients and the control group are presented in Table 2. The genotypic frequencies were tested as well as the condition, which affects them or not, that is, the group each subject belonged to (patients-healthy). As shown, the frequencies of CC, CT, and TT genotypes of rs10046 polymorphism were found to be 0.34, 0.48, and 0.18 for the group of CAD patients and 0.20, 0.48, and 0.32 for the control group, respectively. It was calculated *x* ^2^ = 6.7485 and for two degrees of freedom a correspondence at a probability of *P* = 0.034 was found; that is, the genotypic frequencies differ significantly in the two groups under study. The frequency of C allele was 0.58 and 0.44 for the group of CAD patients and for the control group, respectively, and the frequency of T allele was 0.42 and 0.56 for the group of CAD patients and for the control group, respectively. The C allele shows an increased risk of CAD occurrence (OR = 1.771 and *P* = 0.010). Similar findings were pointed for men with the same statistical significance for the genotypic frequencies (*P* = 0.034) and the C allele risk for CAD (OR = 1.881 and *P* = 0.010); females consisted of small group for patients and controls and did not demonstrate any statistical significance, although the C allele risk had a high odds ratio for the CAD patients (Table 2).

## 4. Discussion

The CYP19A1 gene encodes the enzyme aromatase, which is responsible for the biosynthesis of estrogens. Mutations and other genetic changes in this gene can cause increased or decreased activity of aromatase [3, 4]. More than 80 genetic polymorphisms in CYP19A1 gene have been described. Published data support the correlation of these polymorphisms with the activity of aromatase, sex hormone levels, and estrogen dependent conditions. However, it is unclear what molecular mechanisms are involved [5–9]. Studies have shown that common polymorphisms of the aromatase gene (rs11575899 and rs10046) are associated with estradiol and androgen serum levels in premenopausal and postmenopausal women [17–20].

Sex hormones have been the subject of many studies regarding their correlation with CAD and cardiovascular system. It has been demonstrated that both estrogens and aromatase are produced in vascular tissue, particularly in smooth muscle cells and endothelial cells [10, 11]. Furthermore, premenopausal women have a very lower risk of CAD occurrence compared with men of the same age, while upon menopause a phase of increased risk begins [21]. This suggests that sex hormones play a role in the development of CAD. The lack of sex steroid hormones is considered a critical factor responsible for the changes in CAD risk model associated with menopause and consequently for the increase in the risk of the disease [22, 23]. Although there are data supporting the association between endogenous sex hormones and cardiovascular disease, the results are still contradictory. Various studies have shown a positive correlation between estrogens and the risk of CAD [24–26], while others have opposite results and conclude that estradiol levels do not correlate with the disease risk [27–29]. Studies regarding androgens are also conflicting. One suggests that higher levels of androgens were associated with a reduced degree of carotid artery atherosclerosis [30] while others recorded a positive correlation between testosterone and cardiovascular disease risk [31–33].

A recent study examined genetic variants of CYP19A1 gene for their role in the differences depending on gender with regard to the outcome of cardiovascular disease [12]. In this study, a population with acute coronary syndromes was investigated with a three-year monitoring for mortality and another of hypertensive patients with CAD in comparison with a control group, in which the primary outcome was death, nonfatal myocardial infarction, or nonfatal stroke. The C allele of -81371 C > T polymorphism of CYP19A1 gene was associated with 78% increase in mortality in men and 42% in women. A similar association was also observed in the group of hypertensive patients with CAD, where -81371 C > T polymorphism was associated with 65% increase in mortality from myocardial infarction or stroke in men and 69% decrease in women. This study is the first demonstrating a significant polymorphism interaction of CYP19A1 gene with gender on the outcome of cardiovascular disease [12].

The T allele of the rs10046 polymorphism has been shown either trends of higher levels of estrone and estradiol but no differences for androgens, either lower levels of several androgens but no differences for estrone or estradiol, or higher estrogen to androgen ratios [13]. However, the exact mechanism remains unclear. It is considered that this polymorphism does not act autonomously but in conjunction with other polymorphisms affecting mRNA levels and CYP19A1 gene stability [34]. The rs10046 polymorphism of CYP19A1 gene and its probable association with cardiovascular system have been investigated in two studies, where the association of genetic changes of CYP19A1 gene with the occurrence of hypertension was investigated [14, 15]. The first study recorded a significant correlation of rs10046 polymorphism with blood pressure levels in women and this relationship depended on body mass index, while it was independent of menopausal status [14]. The second study showed that the genotypic risk of rs10046 polymorphism is inversely related to gender in the occurrence of essential hypertension. Systolic and diastolic blood pressure levels were higher in all men bearing T/T genotype compared with those not bearing it. The results were not confirmed in women [15]. The opposite results with regard to gender in these two studies may be explained by the small population samples, possibly by the balance of estrogens/androgens, the levels of which were not measured and by the assumption that the overall result involves other factors as well, the mechanisms of which we do not know.

Our study was performed in two groups with similar risk factors, which were separated by coronary angiographic documentation in patients with CAD and healthy. The results of this study show that rs10046 (C/T) polymorphism of CYP19A1 gene exhibits correlation with CAD. The frequencies of CC, CT, and TT genotypes of rs10046 polymorphism demonstrated significant differences between the group of patients with CAD and the control group (*P* = 0.034) and a significant increased risk was found for patients with C allele (OR 1.771 and *P* = 0.0095), who have an increased probability of manifesting the disease. Similar findings were pointed for men, but not for women, although the C allele had a high odds ratio risk for the CAD patients. The latter result can be explained by the small size group for females. We may conclude that rs10046 polymorphism of CYP19A1 gene is a risk factor for the occurrence of CAD, but without knowing the exact mechanism. To this direction the measure of sex hormones levels would be undoubtedly useful. However, no other studies have been performed on this polymorphism and CAD and, taking into account the multifactors of the disease, it is necessary to significantly increase the sample and the research is continued and confirmed by genome-wide association studies (GWAS) in connection with aromatase activity.

Polymorphisms associated with the occurrence of CAD are risk factors for this disease and our efforts to prevent the onset or progress of the disease must be directed to the people bearing these polymorphisms in their genetic material.

## Figures and Tables

**Table 1:** Clinical characteristics in both groups of subjects (CAD patients/controls).

	Patients (*n* = 123)	Controls (*n* = 66)	*P* value
Age (years)	60 ± 11	58 ± 9	0.080
Male sex, *n* (%)	99 (81)	52 (79)	0.781
Arterial hypertension, *n* (%)	43 (35)	19 (29)	0.389
Smoking, *n* (%)	85 (69)	43 (65)	0.579
Diabetes, *n* (%)	25 (20)	12 (18)	0.723
Family history of CAD, *n* (%)	27 (22)	13 (20)	0.718
Total cholesterol (mg/dL)	185 ± 40	190 ± 17	0.282
HDL cholesterol (mg/dL)	41 ± 10	42 ± 6	0.347
LDL cholesterol (mg/dL)	118 ± 39	120 ± 19	0.508
Triglycerides (mg/dL)	132 ± 53	135 ± 13	0.531
Ancient history of CAD, *n* (%)	23 (19)	0	<0.001

Abbreviations: CAD, coronary artery disease; HDL, high density lipoprotein; LDL, low density lipoprotein; *n*, number of participants. Age, cholesterol, triglycerides, LDL, HDL values are mean ± SD (standard deviation).

**Table 2:** Genotypes and alleles frequencies of rs10046 polymorphism of CYP19A1 gene in patients and controls according to sex.

All participants (*n* = 189)					
	Patients (*n* = 123)	Controls (*n* = 66)	*X* ^²^	OR (95% CI)	*P* value

Genotype	** **	** **	** **	** **	** **
CC, *n* (%)	42 (0.34)	13 (0.20)	** **	** **	** **
CT, *n* (%)	59 (0.48)	32 (0.48)	6.748	** **	0.034
TT, *n* (%)	22 (0.18)	21 (0.32)	** **	** **	** **
Allele	** **	** **	** **	** **	** **
C, %	0.58	0.44	** **	1.771 (1.156–2.175)	0.010****
T, %	0.42	0.56	** **		

Males (*n* = 151)					
** **	Patients (*n* = 99)	Controls (*n* = 52)	*X* ^²^	OR (95% CI)	*P* value

Genotype	** **	** **	** **	** **	** **
CC, *n* (%)	28 (0.28)	7 (0.13)	** **	** **	** **
CT, *n* (%)	51 (0.52)	26 (0.50)	6.769	** **	0.034
TT, *n* (%)	20 (0.20)	19 (0.37)	** **	** **	** **
Allele	** **	** **	** **	** **	** **
C, %	0.54	0.38	** **	1.881 (1.160–3.052)	0.010****
T, %	0.46	0.62	** **		

Females (*n* = 38)					
	Patients (*n* = 24)	Controls (*n* = 14)	*X* ^²^	OR (95% CI)	*P* value

Genotype	** **	** **	** **	** **	** **
CC, *n* (%)	14 (0.58)	6 (0.43)	** **	** **	** **
CT, *n* (%)	8 (0.34)	6 (0.43)	0.915	** **	0.633
TT, *n* (%)	2 (0.08)	2 (0.14)	** **	** **	** **
Allele	** **	** **	** **	** **	** **
C, %	0.75	0.64	** **	1.667 (0.606–4.586)	0.320
T, %	0.25	0.36	** **		

OR: odds ratio and CI: confidence interval.
